# Transcriptional network constituted of CBP, Ku70, NOX2, and BAX prevents the cell death of necrosis, paraptosis, and apoptosis in human melanoma

**DOI:** 10.1038/s41420-021-00417-z

**Published:** 2021-02-26

**Authors:** Liang Ding, Yalei Wen, Xin Zhang, Fang Zhao, Kenao Lv, Jian-hong Shi, Shigang Shen, Xuefeng Pan

**Affiliations:** 1 School of Medicine, Hebei University, Baoding, 071002 China; 2 School of Life Science, Beijing Institute of Technology, Beijin, 100081 China; 3 Central Laboratory, Affiliated Hospital of Hebei University, Baoding, 071002 China; 4 School of Chemistry and environmental Science, Hebei University, Baodin, 071002 China

**Keywords:** Cancer models, Cancer epigenetics

## Abstract

CREB-binding protein (CBP) is an acetyltransferase known to play multiple roles in the transcriptions of genes involving oxidative metabolism, cell cycle, DNA damage checkpoints, and cell death. In this study, CBP was found to positively regulate the expression of Ku70, and both CBP and Ku70 were found to negatively regulate the expression of NOX2, therefore, mitigating the intracellular ROS in human melanoma. Knocking down CBP or Ku70 induced necrotic and paraptotic cell death as indicated by high-level intracellular ROS, cytoplasmic vacuolization, and cell cycle arrest in the S phase. In addition, chromosomal condensations were also observed in the cells proceeding necrotic and paraptotic cell death, which was found to be related to the BAX-associated intrinsic pathway of apoptotic cell death, when Ku70 was decreased either by CBP depletion or by Ku70 depletion directly. Our results, therefore, supported the idea that CBP, Ku70, BAX, and NOX2 have formed a transcriptional network in the prevention of cell death of necrosis, paraptosis, and apoptosis in human melanoma.

## Introduction

CREB-binding protein (CBP) acetyltransferase activates a number of transcription factors, including Nrf2 (nuclear factor erythroid-related factor 2)^1^, MITF^2^, NF-κB^3^, and AP-1 (ref. ^4^). Among which, Nrf2 is involved in monitoring the reactive oxygen species (ROS) and maintaining the redox homeostasis. Nrf2 presents as Nrf2–Keap1 complex in the cytoplasm and enters the nucleus once the Keap1 is modified by ROS. Nrf2 binds to antioxidant response element (ARE) in the promoter regions of multiple genes that encode antioxidant enzymes and phase II detoxification enzymes oxidative metabolism, including heme oxygenase 1, nicotinamide adenine dinucleotide phosphate (NADPH) quinone oxidoreductase 1, and so forth^5^.

The roles of CBP in the Nrf2-regulated gene transcriptions are twofolds, CBP facilitates the assembly of the transcription initiation complex via interacting with Neh4 domain and Neh5 domain in the N-terminal region of Nrf2 (ref. ^6^), while it also works in acetylation of proteins, such as histones in the transcribing chromatin ^7^.

Defective CBP has been found in human diseases, including Rubinstein–Taybi syndrome, tumors (e.g., leukemia, a variety of translocations of CBP genes have been characterized so far), and neurological disorders, such as Huntington’s disease^8^, Alzheimer’s disease^9^, polyglutamine diseases^10^, and spinal and bulbar muscular atrophy^11^. CBP serves as a tumor suppressor by signaling DNA damages and by regulating DNA repair factors in certain tumor conditions^12^. For example, in DNA repairs of double-strand break (DSB) by nonhomologous end joining (NHEJ), CBP has been found to catalyze the acetylation modifications of histones H3 and H4 at a DSB site, to promote the recruitments of Ku70/80 protein to the DSB site through synergizing with SWI/SNF chromatin remodeling complex^13^. In addition, the acetylation modification of the Ku70 by CBP can also be used in controlling its binding with BAX in initiating the mitochondrial pathway of cell apoptosis. During which, BAX is released from the Ku70–BAX complex as a result of the Ku70 acetylation, and the released BAX is then to initiate the mitochondrial pathway of cell apoptosis^14–16^. Acetylation modifications of lysine 539 and 542 in Ku70 release BAX from the Ku70–BAX complex, which leads to cell apoptosis in a BAX- and caspase-dependent fashion in neuroblastoma (NB) cells^16^. However, the roles of CBP in melanoma have not been clearly understood yet.

The roles of ROS in human disease conditions have been well documented^17^. ROS serves as the main source of intracellular redox homeostasis with strong cytotoxicity. Loss of balance between redox molecules and antioxidants in cells could exacerbate the cytotoxic effects of the ROS, leading ultimately to human diseases, including metabolic dysfunctions, neurodegeneration, chronic inflammation, cardiovascular defects, and oncogenesis^16^. ROS can be produced by multiple pathways of metabolism, including glycolysis, gluconeogenesis, lipid metabolism, and ATP or nitric oxide synthesis^18^. Among them, the oxidation of NADPH catalyzed by NADPH oxidase is the main source of ROS production^19^. NADPH oxidase consists of five subunits, including two membrane subunits: gp91^phox^ (NOX2) and p22^phox^, and three cytoplasmic subunits: p47^phox^, p40^phox^, and p67^phox^ (ref. ^20^). It has been reported that epigenetic modifications, such as histone acetylation and deacetylation (histone deacetylase (HDAC)) are essential for the transcriptions of some subtypes of NADPH oxidases, such as NOX2 (ref. ^20^). Decreased transcription of *NOX2* mRNA by applying HDAC inhibitors has been reported in immune cells^20^.

In this study, we have analyzed the roles of CBP in human melanoma A375 by differentially depleting the CBP mRNA. We found that depletion of CBP mRNA upregulated the expression of *NOX2* gene, encoding NOX2 NADPH oxidase, and affected the gene transcriptions of phase II detoxification enzymes via Nrf2–Keap1 pathway, resulting in the rapid elevation of intracellular ROS in melanoma cells. In addition, cytoplasmic vacuolization and cell cycle arrest in S phase were also characterized, and the expression of Ku70 was also decreased. Moreover, the depletion of either CBP or Ku70 caused chromatin condensation and fragmentation, as usually seen in the intrinsic pathway of apoptotic cell death. Further, we also found that the downregulation of *Ku70* gene transcription and translation was much more significant than the mitigation of acetylation modifications of the Ku70 protein by CBP depletion, showing a Ku70 dosage-dependent elevation of BAX in CBP-depleted cells. The BAX then leads to the release of pro-apototic factors, such as cytochrome C from the mitochondria and the activation of the caspases, resulting in the initiation of the intrinsic pathway of apoptosis. Therefore, our results adding up together indicated that CBP, Ku70, NOX2, and BAX have been made up of a transcriptional network in preventing cell death, such as paraptosis and necrosis via NOX2–ROS, and apoptosis via Ku70–BAX–caspases in human melanoma.

## Results

### Depletion of CBP and/or Ku70 inhibited cell growth and caused cell death

**Fig. 1:**
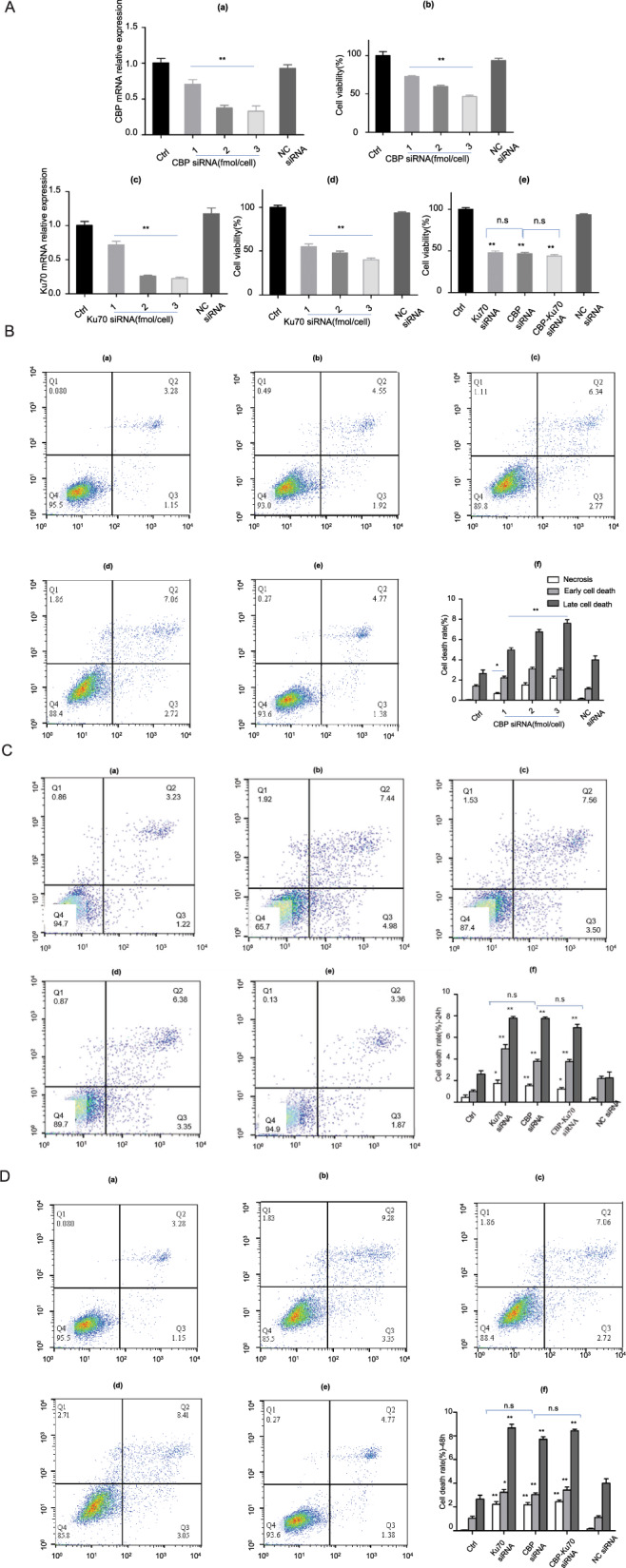
Depletion of CBP and/or Ku70 inhibited cell proliferation and caused cell death. **A** (a) CBP mRNA expression level in human melanoma cells line A375 cells; (b) CCK-8 assay on cell proliferation after transfection CBP siRNA; (c) Ku70 mRNA expression level in human melanoma cells line A375 cells; (d) CCK-8 assay on cell proliferation after transfection Ku70 siRNA; (e) CCK-8 assay on cell proliferation after transfection CBP siRNA and/or Ku70 siRNA. **B** Cell death caused by the depletion of CBP in human melanoma A375 cells in 48 h. (a) Control group. (b) CBP siRNA (1 fmol/cell) group. (c) CBP siRNA (2 fmol/cell) group. (d) CBP siRNA (3 fmol/cell) group. (e) NC siRNA group; (f) the statistical analysis of the cell death. **C** Effects on A375 cell death by co-transfection of CBP siRNA and Ku70 siRNA (24 h). (a) Control group; (b) Ku70 siRNA (70% depletion) group; (c) CBP siRNA (70% depletion) group. (d) Co-transfections of the CBP siRNA and the Ku70 siRNA (60% depletion for CBP and Ku70, respectively); (e) NC siRNA group; (f) the statistical analysis of the cell death rate of A375 cells. **D** Effects on A375 cell death by co-transfection of CBP siRNA and Ku70 siRNA (48 h). (a) Control group; (b) Ku70 siRNA (70% depletion) group; (c) CBP siRNA (70% depletion) group; (d) co-transfections of CBP siRNA and Ku70 siRNA (60% depletion for CBP and Ku70, respectively); (e) NC siRNA group; (f) the statistical analysis of the cell death rate of A375 cells. Data in all bars are expressed as mean ± SEM (*n* = 3). *P < 0.05, **P < 0.01, ^*#*^*P* < 0.05, ^*##*^*P* < 0.01, and n.s denotes *P* > 0.05, when compared with CBP siRNA (by 70% depletion) group.

To understand the roles of CBP and Ku70 in human melanoma cells, we designed and synthesized a set of CBP siRNA and Ku70 siRNA, respectively, and examined their efficiencies in knocking down the CBP mRNA and the Ku70 mRNA in human melanoma A375 cell line by real-time quantitative polymerase chain reaction (PCR). A 2^−△△Ct^ method was used when quantifying the changes of the transcriptions of CBP mRNA in both the control group and the experimental groups (Fig. 1Aa). As shown in Fig. 1Aa, the CBP mRNA has successfully been downregulated in a dosage-dependent manner after the CBP siRNA was transfected into cells by different amounts (Fig. 1Aa).The depletion efficiencies were found to be 20, 40, and 70% when transfections were done by 1, 2, and 3 fmol/cell (*P* < 0.05). By contrast, transfections using negative control siRNA (NC group) failed to decrease the CBP mRNA in the control group (*P* > 0.05).

The effects of CBP depletion on cell proliferation were then analyzed using a CCK-8 assay. The CCK-8 results are presented in Fig. 1Ab. As it can be seen in Fig. 1Ab, depletion of CBP inhibited the cell proliferation of the A375 cells in a siRNA dose-dependent manner. The more the depletion of the CBP mRNA, the more inhibiting effects on cell proliferation of the A375 cells were seen (Fig. 1Ab). By contrast, no significant differences in proliferation inhibition can be noticed between the NC group and the control group (Fig. 1Ab).

Similarly, the Ku70 mRNA in the control group and in the cells differentially depleting Ku70 were also determined by real-time quantitative PCR (Fig. 1Ac). Approximate depletion efficiencies of 30, 70, and 80% were found to be actualized when 1, 2, and 3 fmol/cell of Ku70 siRNA were used in the transfection manipulations, respectively (*P* < 0.05; Fig. 1Ac). The CCK-8 proliferation analysis with the cells depleting Ku70 mRNA are presented in Fig. 1Ad, as shown in Fig. 1Ad, depletion of Ku70 mRNA also inhibited the cell proliferation of the A375 cells in a siRNA dose-dependent manner (Fig. 1Ad).

We then compared the effects of co-transfections of CBP siRNA (70% depletion efficiency) and Ku70 siRNA (70% depletion efficiency) on cell proliferation. The depletion of CBP mRNA by 70% and the depletion of Ku70 mRNA by 70% were actualized by using 3 fmol/cell of CBP siRNA and 2 fmol/cell of Ku70 siRNA, respectively (*P* < 0.05, Fig. 1Aa,Ac). The effects of co-transfections on cell proliferation are presented in Fig. 1Ae. As it can be seen in Fig. 1Ae, the proliferation of A375 cells were found to be significantly inhibited in the CBP siRNA (70% depletion) group, the Ku70 siRNA (70% depletion) group, and the CBP-Ku70 co-transfection (each with a depletion of 60% by PCR quantification), respectively (Fig. 1Ae). No significant differences in the inhibition on A375 cell proliferation between the groups of Ku70 siRNA, the CBP siRNA, and the co-transfection siRNA were noticed (Fig. 1Ae), suggesting that the depletion of CBP and Ku70 individually or simultaneously affected the cell proliferation of A375 cells in a similar fashion.

To understand the mechanism underpinning the cell proliferation inhibition by depleting CBP and Ku70, we have analyzed the cell death induced by the depletion of CBP by flow cytometry, we found a positive correlation between the necrosis, early cell death, and late cell death and the differential depletion of CBP (Fig. 1B). In addition, we also compared the cell death induced by depleting CBP and Ku70, and found no significant difference between the two sets of analysis (Fig. 1C, D), which is in good agreement with the Cell Counting Kit-8 (CCK-8) analysis.

### Depletion of CBP and/or Ku70 caused significant cytoplasmic vacuolization and ROS accumulation

**Fig. 2:**
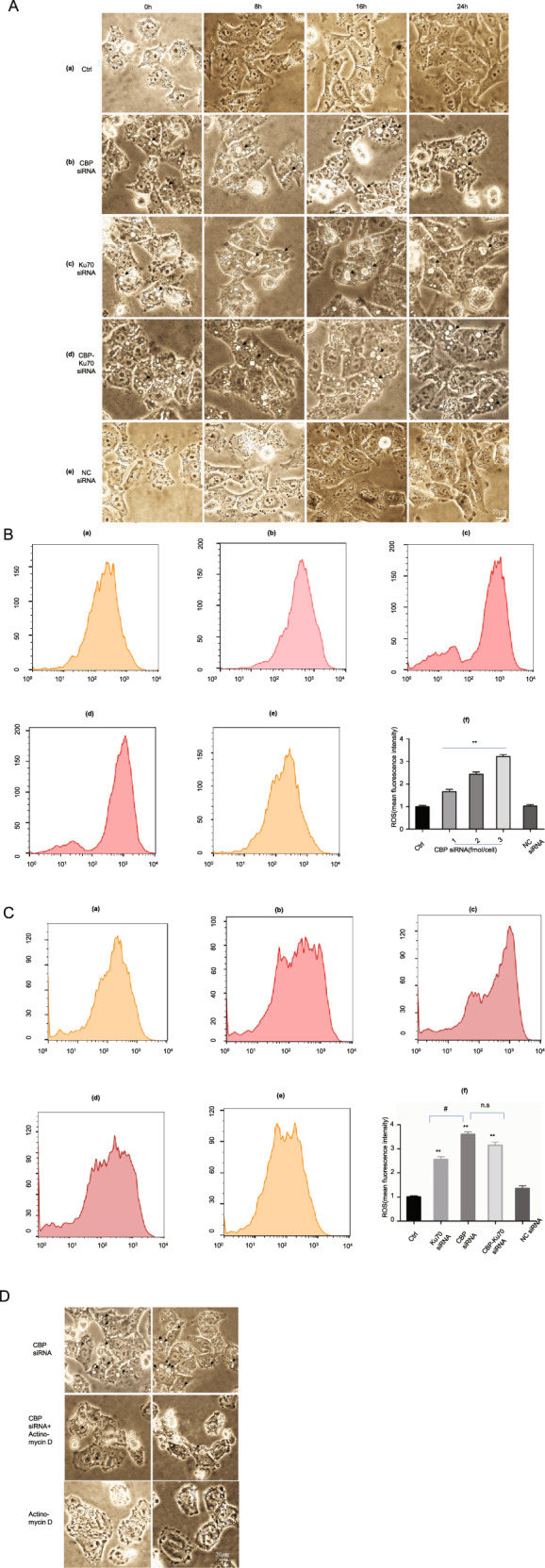
Depletion of CBP and/or Ku70 induced cytoplasmic vacuolization and elevated the intracellular ROS. **A** Cytoplasmic vacuolization (cell morphology under a light microscope (×400)). (a) Control group; (b) CBP siRNA (70% depletion) group; (c) Ku70 siRNA (70% depletion) group; (d) co-transfections of CBP siRNA and Ku70 siRNA (60% depletion for CBP and Ku70, respectively); (e) NC siRNA group. Cytoplasmic vacuolization appeared 0 h after CBP siRNA transfection (black triangle); **B** ROS generation was measured by the DCF fluorescence intensity. (a) Control group. (b) CBP siRNA (1 fmol/cell) group. (c) CBP siRNA (2 fmol/cell) group. (d) CBP siRNA (3 fmol/cell) group. (e) NC siRNA group. (f) Quantitative analysis of ROS generation. **C** Effects of co-transfection of CBP siRNA and Ku70 siRNA on the ROS accumulation in A375 cells. (a) Control group. (b) Ku70 siRNA (70% depletion) group. (c) CBP siRNA (70% depletion) group. (d) Co-transfections of CBP siRNA and Ku70 siRNA (60% depletion for CBP and Ku70, respectively) group. (e) NC siRNA group; (f) quantitative analysis of ROS generation. Data as shown in the bars are expressed as mean ± SEM (*n* = 3). *P < 0.05, *P* < 0.01, ^*#*^*P* < 0.05, ^*##*^*P* < 0.01, and n.s denotes *P* > 0.05, when compared with CBP siRNA (70% efficiency) group. **D** The cytoplasmic vacuolization induced by ROS accumulation is actinomycin D dependent in human melanoma A375. CBP siRNA (70% depletion) group. CBP siRNA (70% depletion) and actinomycin D (5 µg/ml) group. Actinomycin D (5 µg/ml) group.

The morphological changes of the cells affected by the transfections of either CBP siRNA (by 70% depletion) or Ku70 siRNA (by 70% depletion) were also observed under a light microscope (Fig. 2A). As shown in Fig. 2A, significant cytoplasmic vacuolization was found to be associated with the proliferation of A375 cells depleting CBP mRNA and/or Ku70 mRNA (Fig. 2A).

To understand the cause underpinning the cytoplasmic vacuolization, we have further examined the alterations of the intracellular ROS in the cells depleting CBP and Ku70 by flow cytometry (Fig. 2B). As shown in Fig. 2B, a significant elevation in intracellular ROS levels was found to be associated with the CBP depletion by a siRNA dosage-dependent manner (Fig. 2B). We then further compared the ROS production in the cells individually depleting CBP (by 70% depletion), Ku70 (by 70% depletion), and simultaneously depleting both CBP (by 60% depletion) and Ku70 (by 60% depletion; Fig. 2C). The results are presented in Fig. 2C. As it can be seen in Fig. 2C, the intracellular ROS in A375 cells depleting Ku70 was indeed elevated (Fig. 2C). But it was elevated slightly less than the intracellular ROS in the cells depleting CBP (Fig. 2C). Interestingly, the intracellular ROS in the cells simultaneously depleting Ku70 and CBP showed similar elevation to the intracellular ROS in the cells depleting CBP mRNA only (Fig. 2C), suggesting that CBP and Ku70 may work together in repressing the intracellular ROS production (Fig. 2C).

Paraptotic cell death and necrotic cell death do not show apoptotic characteristics of pyknosis, DNA fragmentation, chromatin condensations, and caspase activations^21^, but share common features of cytoplasmic vacuolization^22^. The cytoplasmic vacuolization shared by them could be distinguished by a different response to actinomycin D. Actinomycin D inhibits gene transcription by binding DNA at the transcription initiation complex and preventing elongation of RNA by RNA polymerase, which is expected to mitigate the cytoplasmic vacuolization in the parapototic cell death. In this work, we found that the cytoplasmic vacuolization associated with ROS accumulation can be decreased by actinomycin D (Fig. 2D), suggesting that the cytoplasmic vacuolization associated with the CBP depletion or the Ku70 depletion was at least an indication of the presence of paraptotic cell death (Fig. 2D).

### Increased ROS by CBP depletion was due partially to the malfunctions of the transcriptions of the genes in the Nrf2–Keap1 pathway

**Fig. 3:**
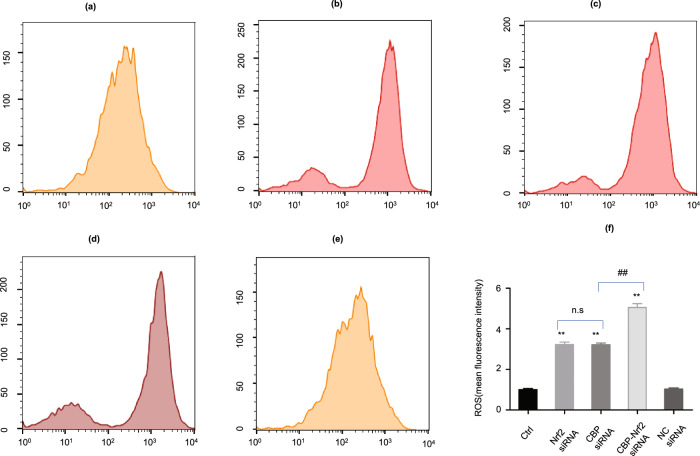
Effects of co-transfection of CBP siRNA and Nrf2 siRNA on the ROS accumulation in A375 cells. **a** Control group; **b** Nrf2 siRNA(70% depletion); **c** CBP siRNA(70% depletion); **d** co-transfections of CBP siRNA and Nrf2 siRNA (the dose of siRNA applied equals to 70% depletion of CBP and Nrf2, respectively); **e** NC siRNA group; **f** measurements of the ROS by the DCF fluorescence intensity. Data as shown in the figures were expressed as mean ± SEM (*n* = 3). *P < 0.05, **P < 0.01, ^*#*^*P* < 0.05, ^*##*^*P* < 0.01, and n.s denotes *P* *>* 0.05.

To understand if the increased ROS may or may not be due to the failure of CBP-associated transcriptions of genes encoding intracellular antioxidant enzymes and the phase II detoxification enzymes in the Nrf2–Keap1 pathway^23,24^. CBP and Nrf2 siRNAs were co-transfected with the melanoma cells by a dose roughly equal to 70% depletion of either CBP mRNA or Nrf2 mRNA. The yields of ROS in the cells depleting either CBP mRNA, Nrf2 mRNA alone, or depleting both CBP mRNA and Nrf2 mRNA were measured by flow cytometry, and the results are presented in Fig. 3. As it can be seen in Fig. 3, the ROS levels in the cells depleting CBP and in the cells depleting Nrf2 mRNA were similar, no significant differences can be seen between them (Fig. 3). However, a synthetic effect of the ROS accumulation by simultaneously depleting both CBP mRNA and Nrf2 mRNA can be clearly seen, when compared with the ROS accumulated in the cells depleting either CBP mRNA or Nrf2 mRNA individually, suggesting that the enhancement of ROS level by depleting CBP mRNA was not purely due to the failure of CBP functions in the gene transcriptions of the intracellular antioxidant enzymes and the phase II detoxification enzymes in the Nrf2–Keap1 pathway, rather our results suggested that the depletion of CBP mRNA may also enhance the ROS production in an Nrf2–Keap1 pathway-independent manner (Fig. 3).

### Depletion of either CBP or Ku70 increased expression of NOX2

**Fig. 4:**
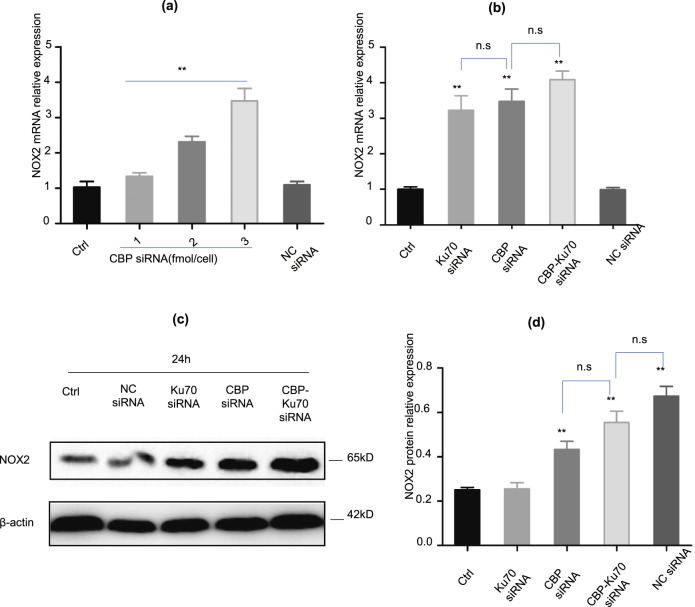
Depletion of CBP and/or Ku70 enhanced the expression of NOX2. **a** The NOX2 mRNA level evaluated by RT-PCR; **b** the transcription of NOX2 when depleting Ku70, and simultaneously depleting both CBP and Ku70; **c** increased NOX2 protein in the cells depleting CBP and/or Ku70 measured by western blotting (the expression was compared to human β-actin as a gene for normalization); **d** The NOX2 protein level evaluated using western blotting, when depleting CBP or Ku70 individually or simultaneously. Data as shown in the figures were expressed as mean ± SEM (*n* = 3). *P < 0.05, **P < 0.01, ^*#*^*P* < 0.05, ^*##*^*P* < 0.01, and n.s denotes *P* > 0.05.

To further understand the cause of the elevated ROS production in the cells depleting CBP, we then carried out the reverse transcription-quantitative PCR (RT-qPCR) quantification of the *NOX2* mRNA in the A375 cells depleting CBP (Fig. 4a). As shown in Fig. 4a, differential depletion of CBP upregulated the gene transcription of *NOX2* in a dose-dependent manner (Fig. 4a).

To further investigate the mechanism of ROS accumulation in Ku70-depleted A375 cells, we also performed RT-qPCR analysis on the transcription of NOX2 mRNA in the cells depleting Ku70 (Fig. 4b). As shown in Fig. 4b, NOX2 mRNA expression was indeed upregulated in the Ku70-depleted cells. Interestingly, we found that the upregulation of NOX2 in the cells depleting CBP or Ku70 was similar to each other (Fig. 4a, b), suggesting that CBP and Ku70 are presumably playing similar roles in the transcription of the *NOX2* gene. Finally, we also confirmed the increase in the protein level of NOX2 by western blotting (Fig. 4c, d). As shown in Fig. 4c, d, the expressions of NOX2 protein were indeed increased (Fig. 4c, d).

### Depletion of CBP/Ku70 induced chromatin condensation

**Fig. 5:**
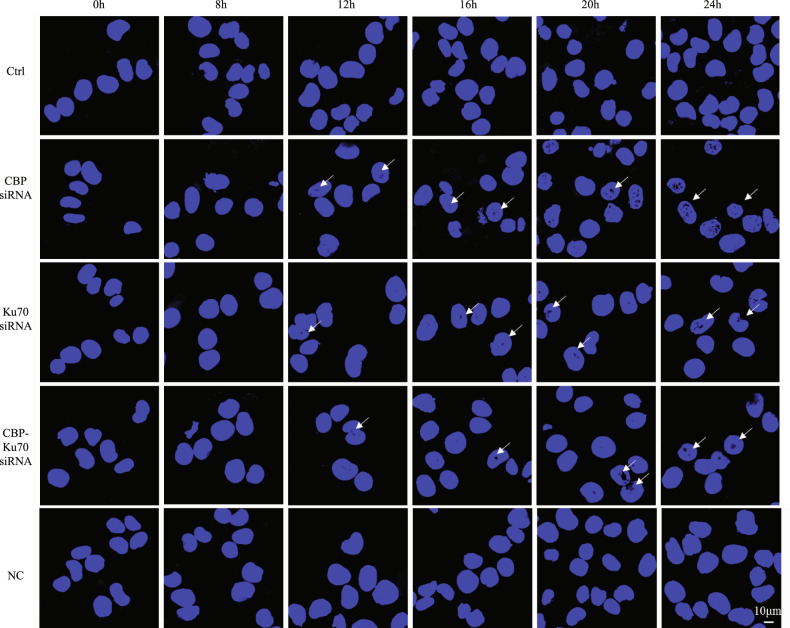
Depletion of CBP and/or Ku70 caused chromatin condensations in human melanoma A375 cells. Nuclear morphology under confocal fluorescence microscopy (×1000). Chromatin condensation appeared 16 h after CBP siRNA transfection (white arrow). Experimental grouping: CBP siRNA (70% depletion) group, Ku70 siRNA (70% depletion) group, and CBP-Ku70 co-transfection of siRNA (60% depletion for CBP and Ku70, respectively) group.

Since we observed a significant increase in the late cell death are associated with the cells depleting either CBP and/or Ku70, we then attempted to know if apoptotic cell death was also existing. To this end, we stained the cells depleting CBP and/or Ku70 (as aforementioned) with DAPI to look at the possible chromatin condensation known to be associated with apoptotic cell death. We visualized the DAPI-stained nucleus under a confocal microscope (Fig. 5). As shown in Fig. 5, vigorous chromatin condensations were indeed seen in the cells depleting either CBP or Ku70 individually, or depleting both CBP and Ku70 simultaneously (Fig. 5), suggesting that apoptotic cell death was indeed being induced by the depletion of CBP and/or Ku70 (Fig. 5).

### Depletion of CBP downregulated the expression of Ku70 and caused apoptosis

**Fig. 6:**
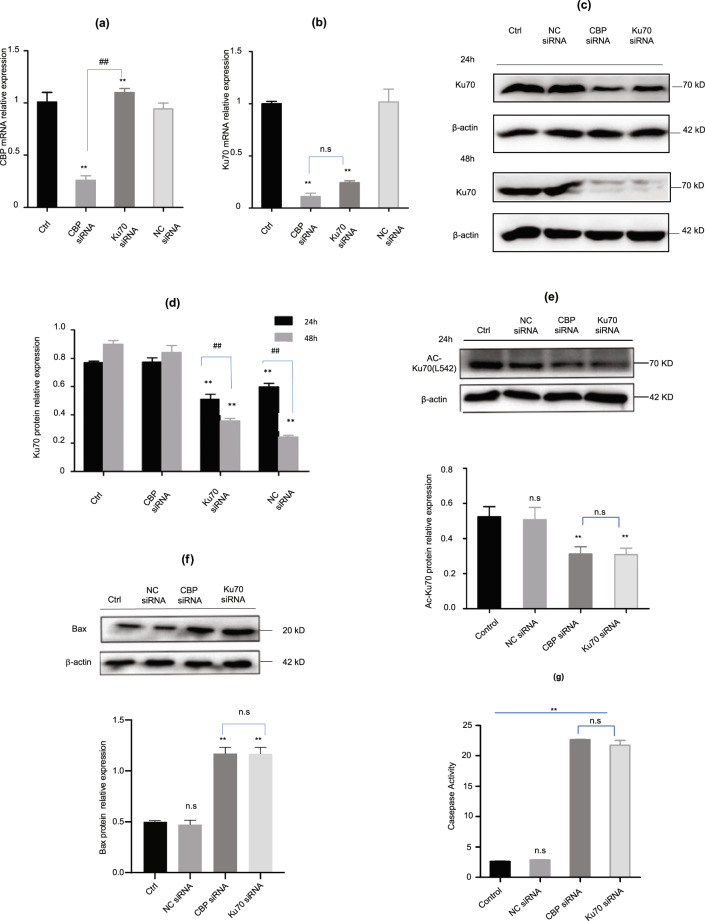
Depletion of CBP downregulated the expression and acetylation modification of Ku70. **a** Changes in CBP mRNA in melanoma cells depleting either Ku70 or CBP, respectively; **b** changes in Ku70 mRNA in melanoma cells depleting either Ku70 or CBP, respectively; **c** changes in the level of Ku70 protein molecules in melanoma cells depleting either Ku70 or CBP for 24 and 48 h, respectively; **d** quantifications of Ku70 protein levels in melanoma cells when depleting either Ku70 or CBP based upon the western blotting in **c**; **e** western blotting analysis on the Ku70(L542) (modified by acetylation at L542) in melanoma cells when depleting either Ku70 or CBP for 24 h; **f** Western blotting analysis on the BAX in melanoma cells when depleting either Ku70 or CBP for 24 h; **g** the elevated caspase-3 activity when depleting either CBP or Ku70. The caspase-3 activity was measured using the chromogenic substrate Ac-DEVD-pNA when the corresponding depletion was conducted for 24 h. The bar represents the increase of the absorbance of pNA produced as a function of caspase-3 cleavages. Data as shown in the figures were expressed as mean ± SEM (*n* = 3). *P < 0.05, **P < 0.01, ^*#*^*P* < 0.05, ^*##*^*P* < 0.01, and n.s denotes *P* > 0.05.

To understand the possible relations between CBP and Ku70 in the elevation of intracellular ROS, we then determined the expression of Ku70 in the cells depleting CBP, Ku70, and CBP–Ku70, respectively. The results are presented in Fig. 6. As shown in Fig. 6, the depletion of the Ku70 alone didn’t affect the transcription of CBP mRNA (Fig. 6a), while the depletion of CBP did significantly downregulate both the mRNA and the protein of Ku70 (Fig. 6b–d), indicating that CBP regulates the expression of Ku70, not vice versa. Further, we also detected the acetylation modification of the Ku70 in the cells depleting CBP (Fig. 6e), we found that the acetylated Ku70 was reduced in the cells depleting CBP mRNA (Fig. 6e), but the reduction was rather similar to that in the cells depleting Ku70 mRNA individually (Fig. 6e), suggesting that the reduction of the acetylated Ku70 in the cells depleting CBP was mainly dependent on the reduced expression of Ku70 rather than the reduced acetylation capacity of the CBP (Fig. 6e). We then further analyzed the BAX and caspase-3 in the cells depleting CBP (Fig. 6f, g). We found that both BAX expression (Fig. 6f) and caspase-3 activity (Fig. 6g) were increased. These results suggested that CBP has played dual roles by regulating the Ku70 expression and by the acetylation modification of Ku70.

### Depletion of CBP and Ku70 arrested cell cycle in S phase

**Fig. 7:**
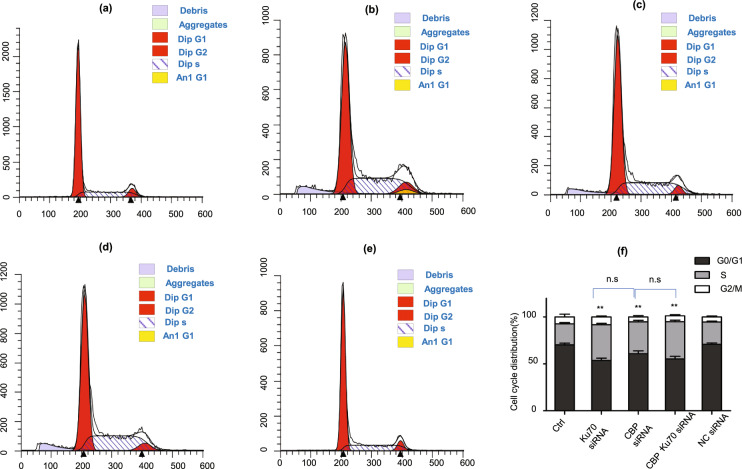
Depletion of CBP and/or Ku70 caused cell cycle arrest. **a** Control group. **b** Ku70 siRNA (70% depletion) group; **c** CBP siRNA (70% depletion) group; d co-transfections of CBP siRNA and Ku70 siRNA (60% depletion for CBP and Ku70, respectively). **e** NC siRNA group; **f** cell cycle of different groups of A375 cells. Data as shown in the figures were expressed as mean ± SEM (*n* = 3). *P < 0.05, **P < 0.01, ^*#*^*P* < 0.05, ^*##*^*P* < 0.01, and n.s denotes *P* > 0.05.

To investigate if the depletion of CBP and/or Ku70 affected the cell cycle of the melanoma cells, we have further analyzed the cell cycle of the melanoma cells depleting CBP and/or Ku70 by flow cytometry. The results are presented in Fig. 7a–f. As shown in Fig. 7a–f, the cell numbers of A375 cells depleting either CBP or Ku70 showed no significant difference to those of the NC siRNA and control groups in the G2/M phase of the cell cycle. However, numbers of S phase cells in the CBP and/or Ku70-depleted groups were significantly increased compared to those in the NC siRNA group. These results clearly indicated that depletion of either CBP or Ku70 in melanoma cells arrested the cell cycle in the S phase, conceivably due to the elevated ROS made by the increased NOX2 (Fig. 7a–f).

## Discussion

Human melanoma is a highly invasive and fatal disease, intervention as early as possible has thought to be essential for an ideal prognosis^25^. It has been well documented that the proliferation of melanoma cells may be challenged by a high level of ROS produced by multiple pathways of metabolic processes, including mitochondria, melanosomes, NOX, and NOS^26^. ROS could affect melanoma proliferation in dual ways, excessive intracellular ROS may increase DNA damage and chromosomal degradation, leading to genetic instability, or activate antitumor signaling pathways, initiating oxidative stress-induced tumor cell death, or promote tumor reductive signal transduction and enhance cell proliferation and survival^27^.

The Nrf2–Keap1 pathway is found to be a key mechanism of the ROS monitor. Of which, CBP worked with Nrf2 in transcribing the genes encoding the intracellular antioxidant enzymes and the phase II detoxification enzymes^28–30^ When the ROS level was raised, Nrf2 disassociates with Keap1 and enters the nucleus to bind its specific ligand Maf, forming a heterodimer, Nrf2–Maf. This heterodimer then binds the ARE with the assistance of CBP to activate gene transcriptions of antioxidative enzymes and phase II detoxification enzymes as aforementioned^23^.

In this study, we have found that CBP has not only been used in the transcriptional activation of the Nrf2 target genes through interacting with the CREB bound in the CRE *cis*-acting element (cAMP response element; as previously reported), but also played opposite roles in repressing the expression of *NOX2* and in enhancing the expression of *Ku70*. Depletion of CBP promoted the *NOX2* expression while downregulated the Ku70 expression, leading to significant cell death showing server cytoplasmic vacuolization, increased ROS accumulation, cell cycle arrest in the S phase, and chromosomal fragmentation in melanoma cells.

**Fig. 8:**
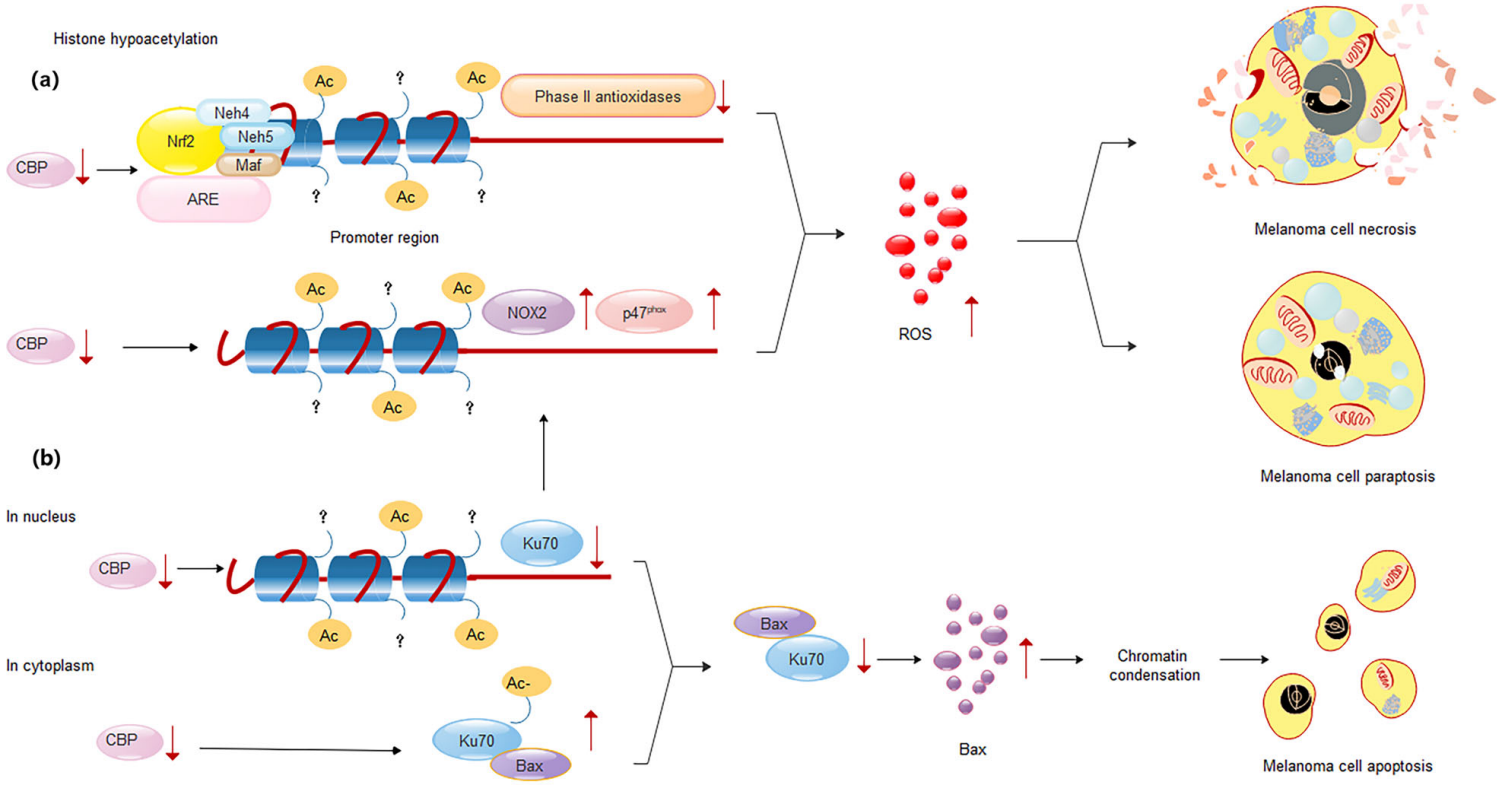
The gene regulatory network composed of CBP, Ku70, and NOX2 for the prevention of necrosis, paraptosis, and apoptosis in human melanoma. **a** The transcriptions of the CBP, Ku70, and NOX2 are responsible for ROS-related necrosis and paraptosis; **b** transcriptions of the CBP, Ku70, and Bax are responsible for the apoptosis.

A working model illustrating the roles of CBP and Ku70 maintaining a balanced ROS-associated redox in melanoma cells is presented in Fig. 8. In Fig. 8, CBP was proposed to play roles in producing antioxidants through the Nrf2–Keap1 pathway and also in switching off the expression of *NOX2* through working with Ku70, offering the melanoma cells an appropriate redox condition for proliferation (Fig. 8). In such a process, CBP was also required by the expression of Ku70, which produces enough Ku70 to form the Ku70–BAX complex, to switch off the BAX-associated apoptotic cell death (the intrinsic pathway of apoptosis, Fig. 8). Obviously, CBP, Ku70, NOX2, and CBP, Ku70, and BAX are organized into lines of the regulatory network in balancing the oxidative stress for melanoma cells to proliferate (Fig. 8).

Ku70 and Ku80 have long been known to play roles in NHEJ repair of DNA DSBs^31,32^. During which, CBP/p300 promotes the recruitments of Ku70 and Ku80 to the DNA DSB site^33^. Recently, the role of Ku70 in controlling apoptotic cell death has been found in NB cells. Ku70 binds to BAX to form the Ku70–BAX complex in the cytoplasm, which is found to be critical in controlling the intrinsic pathway of apoptosis. CBP could abolish the BAX binding capacity of Ku70 by acetylation modification on Ku70, releasing the BAX for initiating apoptosis via mitochondria^13^. In addition, similar acetylation modification of Ku80 by CBP was also found to be required by *COX2* transcription^34^. Moreover, Ku80 was found to be able to inhibit melanoma cell proliferation and induce apoptosis; however, similar roles of Ku70 in melanoma cells has remained unknown^35^. In this work, we have revealed two roles of Ku70 in melanoma cells: negatively regulate the transcription of *NOX2* and positively control the apoptosis via forming Ku70–BAX complex. We clearly demonstrated that knocking down Ku70 in melanoma cells have elevated the ROS level, arrested cell cycle in the S phase, inhibited the proliferation of melanoma cells, and induced cell death, such as paraptosis, necrosis, and apoptosis (Fig. 8).

### Summary

In this study, we found for the first time that CBP and Ku70 have been utilized in the negative regulation of *NOX2* expression. Knocking down either CBP or Ku70 results in enhanced expression of *NOX2*, and increased level of intracellular ROS, which is responsible for cytoplasmic vacuolization, cell cycle arrest in S phase, necrosis, and parapotic cell death. In addition, CBP also functions in the expression and the acetylation modification of Ku70. Knocking down CBP increased the level of BAX in the cytoplasm, leading to the release of pro-apoptotic factors from mitochondria and the activations of caspases for the induction of apoptosis. In summary, we found in this work that CBP, Ku70, NOX2, BAX, and caspase have been organized into a transcriptional network in balancing the ROS-related intracellular oxidative environments, which benefits the proliferation of melanoma cells by avoiding the necrosis, paratosis and apoptotic cell death.

## Materials and methods

### Materials

Dulbecco’s modified Eagle’s medium (DMEM), penicillin/streptomycin, and fetal bovine serum (FBS) were purchased from Gibco-BRL (Gaithersburg, MD, USA). ExFect^TM^2000 transfection reagent was purchased from Vazyme Biotech (Nanjing, China). PrimeScript^TM^ RT reagent kit (Perfect Real-Time) and SYBR Premix Ex Taq^TM^ II (Tli RNaseH Plus) were purchased from Takara Biotechnology Co. Ltd (Dalian, China). Trizol reagent was purchased from Invitrogen (Carlsbad, CA, USA). RIPA lysis buffer with a protease inhibitor phenylmethanesulfonyl fluoride (PMSF), PVDF membrane, BCA protein assay kit, and ECL Western Blotting Substrate Kit were purchased from Solarbio (Beijing, China). Ku70 (acetyl L542) antibody (Rabbit IgG) was purchased from SAB (MD, USA). BAX antibody (Rabbit IgG) was purchased from ABcam (Cambridge, MA, USA). NOX2 antibody (Rabbit IgG), Ku70 antibody (Rabbit IgG), β-actin antibody (Rabbit IgG), and Goat Anti-Rabbit IgG (H + L) were purchased from ABways (Shanghai, China). Kit for analyzing caspase-3 activity was purchased from Beyotime Biotechnology Co. Ltd (Nantong, China). CCK-8 was purchased from Dojindo Molecular Technologies, Inc. (Kumamoto, Japan). Annexin V–propidium iodide (PI) apoptosis detection kit was purchased from Vazyme Biotech (Nanjing, China). Kits for analyzing the cell cycle and apoptosis were purchased from Beyotime Biotechnology Co. Ltd (Nantong, China). Antifade Mounting medium with DAPI was purchased from Solarbio (Beijing, China). Actinomycin D was purchased from the Sigma Chemical Co. (St. Louis, MO, USA). siRNAs (listed in Table 1) were designed by use of an online tool (http://biodev.extra.cea.fr/DSIR/DSIR.html), and examined for the sequence specificity by running Basic Local Alignment Search Tool [National Center for Biotechnology Information (NCBI)], and synthesized by Sangon Biotech (Shanghai, China). Primers used in this study are listed in Table 2, they were also synthesized by Sangon Biotech (Shanghai, China).

**Table 1:** The sequences for siRNA.

siRNA		Sequences
CBP siRNA	Forward	GGCGAAUGACAGCACAGAUTT
	Reverse	AUCUGUGCUGUCAUUCGCCTT
Ku70 siRNA	Forward	GCAGUGGACCUGACAUUGCTT
	Reverse	GCAAUGUCAGGUCCACUGCTT
Nrf2 siRNA	Forward	GGAUUUGAUUGACAUACUUTT
	Reverse	AAGUAUGUCAAUCAAAUCCTT
NC siRNA	Forward	UUCUCCGAACGUGUCACGUTT
	Reverse	ACGUGACACGUUCGGAGAATT

**Table 2:** The primer sequences for RT-PCR.

Gene		Primer DNA sequences
Human *CBP*	Forward	ATTGATAACGAGGTCCCTACCC
	Reverse	GCTCCGTCTTCATTTCCAG
Human *Ku70*	Forward	TCTCAAGCCTCCTCCAATA
	Reverse	GTCATCCAACTCTTCTTCCT
Human *NOX2*^20^	Forward	GCAGCCTGCCTGAATTTCA
	Reverse	TGAGCAGCACGCACTGGA
Human *β-actin*	Forward	CCTGGCACCCAGCACAAT
	Reverse	GGGCCGGACTCGTCATAC

### Cell culture

The human malignant melanoma A375 cell line (A375-P) was purchased from the Institute of Basic Medical Sciences, Chinese Academy of Medical Sciences (Beijing, China). The A375 cells were cultured in DMEM supplemented with 10% FBS, 100 U/ml penicillin, and 100 mg/ml streptomycin with 5% CO_2_ at 37 °C in a humidified incubator^36,37^. All manipulations were carried out by strictly following the experimental guidelines and ethical regulations of Hebei University (Baoding, China).

### Transfection of siRNA

Transfections of siRNA into melanoma cells were performed by use of an ExFect^TM^2000 Transfection Reagent. The cells were seeded by 1 × 10^**5**^ cells/well into the wells in six-well plates. A375 cells were transfected with siRNAs treated with the transfection reagent when the cells grow ~60–80% in the wells. The siRNAs were diluted into different concentrations using a serum-free medium and mixed up with an equal volume of transfection reagents before use^38^. Melanoma cells were subdivided into control groups, NC siRNA group, and experimental groups. Transfections of the experimental group with siRNA were carried out by a concentration of 1, 2, and 3 fmol/cell, respectively, (the depletion efficiencies were scored to be equal to 70% of the Ku70 mRNA and the CBP mRNA to that in the control group, respectively, for the single transfections with either CBP siRNA or Ku70 siRNA, and to 60% of CBP mRNA and Ku70 mRNA to that in the control group for co-transfections of the CBP siRNA and Ku70 siRNA, respectively). The transfected A375 cells were incubated for 24 h before analysis. The depletion efficiencies were scored by RT-qPCR following the protocol from the manufacture.

### RNA extraction and real-time quantitative reverse transcription PCR

Total RNA molecules were prepared using Trizol reagent by following the standard protocol from the manufacturer^39^. RNA samples were reverse transcribed into cDNA using a PrimeScript^TM^ RT reagent kit with gDNA eraser (TaKaRa Biotechnology Co. Ltd) in a total volume of 20 µl by following the manufacturer’s protocol^40^. The cDNA molecules obtained by the above procedure were used as amplification templates, and the expression levels of the target genes were measured by real-time quantitative PCR. The PCR was quantified according to the instructions of the SYBR Premix Ex Taq TM II (Tli RNaseH Plus) kit (Takara Biotechnology Co. Ltd) in a CFX96 Real-Time PCR Detection System. The amplification steps were as follows: steps are (i) at 95 °C for 30 s; (ii) 40 cycles at 95 °C for 5 s; (iii) at 55 °C for 30 s; and (iv) extension at 72 °C for 30 s. β-actin was used as an endogenous reference, and each sample was normalized to its β-actin content. Results are represented as the refractive induction using the 2^−△△Ct^ method.

### Protein extraction and western blot analysis

Cells were harvested and lysed using a RIPA lysis buffer (Solarbio, Beijing, China) with PMSF (Solarbio, Beijing, China) by following the manufacturer’s instruction. Protein concentrations were determined by using a BCA protein assay kit (Solarbio, Beijing, China). An equal amount of protein was loaded onto different wells on an SDS–PAGE gel (containing 7.5% SDS), and electrophoresis was performed at a constant voltage of 120 volts. The separated proteins were then transferred to a PVDF membrane (Solarbio, Beijing, China) at a constant current of 280 amps. The membrane was blocked with 5% w/v skim milk for 1 h at room temperature. The membrane was then incubated in solutions containing either NOX2 antibody (1:1000 dilution), Ku70 antibody (1:500 dilution), or β-actin antibody (1:1000 dilution) at 4 °C for an overnight. Goat anti-rabbit IgG (H + L) (1:1000 dilution) coupled with the corresponding horseradish peroxidase was incubated with an ECL Western Blotting substrate kit. The protein bands were observed and repeated three times for each of the samples. The ImageJ software was used to quantify the bands and to normalize the target protein level to β-actin.

The relative expression of the target protein = the target protein ID value/the internal reference ID value.

### CCK-8 assay

Melanoma A375 cells of 5 × 10^3^ cells/well were seeded in each well in a 96-well plate and incubated for 24 h. Then CCK-8 solution was added to each well, and the cells further incubated at 37 °C for 60 min. Cell viability was measured by the absorbance at 450 nm on a microplate reader^37^. The cell proliferation rates were calculated by use of the following equation:

Cell proliferation rates = A (transfection group)/A (control group) × 100%.

#### Detection of intracellular ROS

The ROS in the melanoma A375 cells were determined using a ROS assay kit purchased from Beyotime Institute of Biotechnology (Haimen, Jiangsu, China). The fluorescent probe used in the assay was 2′, 7′-dichlorofluorescein diacetate (DCFH-DA). DCFH-DA could diffuse into cells and be oxidized by ROS and converted into DCFH, which can be further oxidized to a fluorescent product DCF by ROS.

For the determination of the intracellular ROS in the transfected A375 cells, 10 mM DCFH-DA was diluted to a final concentration of 10 μM with a serum-free DMEM medium. In 24 h of siRNA transfection, the original cell culture medium was discarded and 1 ml of DCFH-DA solution (10 μM) was added into each well, and the cells were further incubated at 37 °C for 30 min and then washed three times using DMEM medium^41^. The ROS of the cell suspension was analyzed at excitation wavelength 488 nm and emission wavelength 525 nm, using FACS can flow cytometer (BD Biosciences).

### Cell death assay

Cell death caused by the transfections of CBP siRNA and Ku70 siRNA was analyzed by use of a kit containing PI and Annexin V-FITC. Firstly, the transfected cells were cultivated at 37 °C with 5% CO_2_ for 24 and 48 h, respectively, and then they were harvested and digested into single-cell suspensions using trypsin digestion solutions (0.25%, without phenol red; Solarbio, Beijing, China). The single cells were washed twice with cold PBS and resuspended in 100 μl of 1× binding buffer (supplied by the manufacturer). The cells were double-stained in dark at room temperature by mixing up with 5 μl o PI staining solution and 5 μl of Annexin V-FITC for 10 min, and resuspended in 400 μl of 1× binding buffer (supplied by the manufacturer). The cell death was analyzed within 1 h by a FACS Calibur instrument (FACS Calibur, BD, USA; the excitation wavelength was 488 nm, and the Annexin-FITC and PI were measured in FL1 channel, and in FL2 or FL3 channel, respectively) ^42^.

### Observation of cytoplasm vacuolization

The human melanoma A375 cells were seeded into the wells of a six-well plate by 1 × 10^5^ cells per well and cultivated for 24 h (as described in the previous). Transfections for knocking down CBP mRNA and Ku70 mRNA individually or simultaneously in the melanoma A375 were conducted, using CBP siRNA and Ku70 siRNA (as described in the “Transfection of siRNA” section). The transfected cells were further cultured for 24 h, and the morphological changes of the cells were observed by using an optical microscope at an interval of 8 h.

For the observations of the effects of actinomycin D on cytoplasm vacuolization in the cells transfected with CBP siRNA, melanoma cells were seeded in a six-well plate by 1 × 10^5^ cells per well and cultivated for 24 h. CBP siRNA (70% efficiency of depletion) and actinomycin D (5 μg/ml) were added simultaneously, and the cell cytoplasm vacuolization was observed under a light microscope in 24 h of transfection.

### Chromatin condensation analysis

The chromatin condensation was analyzed using DAPI staining^43^, specifically, 1 × 10^5^ melanoma A375 cells were seeded into a well in a six-well plate placed with a sterilized glass coverslip in the well, and cultivated in the presence of 5% CO_2_ gas at 37 °C for 24 h. The cells were subdivided into control group, CBP siRNA group (with 70% efficiency of CBP mRNA depletion), Ku70 siRNA group (with 70% efficiency of Ku70 mRNA depletion), CBP–Ku70 siRNA co-transfection group (with 60% efficiencies of CBP mRNA and Ku70 mRNA depletion, respectively), and NC siRNA group. The cells of each group were processed using 1 ml fix solution containing 4% paraformaldehyde (Beyotime Biotechnology, Nantong, China) for 10 min, when the cultivations were at 0, 8, 12, 16, 20, and 24 h, respectively. The fixed cells in each well were washed twice with PBS, and then further processed by adding 1 ml of Immunostaining Permeabilization Buffer containing Triton X-100 (Beyotime Biotechnology, Nantong, China) into the wells for 10 min at room temperature. The cells were then stained in 10 μl of Antifade mounting medium with DAPI (Solarbio, Beijing, China). The chromatin condensation was analyzed under a fluorescence microscope (Olympus, BX61; ×1000).

### Cell cycle analysis

A375 cells were seeded by 1 × 10^5^ into each well containing 2 ml of DMEM medium in a six-well plate and cultivated for 24 h to allow cells to adhere. After 8 h of transfection, fresh DMEM was added to replace the used DMEM medium, and further cultivated for 24 h. Cells were then harvested and trypsinized (trypsin digestion solutions, 0.25% phenol red free) into a single-cell suspension. The single cells were washed twice with PBS and then fixed using 70% ethanol at 4 °C overnight. The fixed cells were then washed once with PBS and then 1 ml of PI staining reagent (containing 50 mg/ml PI and 1 mg/ml RNase in 1 ml of sodium citrate buffer, pH 7.4) was added and kept in dark at room temperature for 30 min (ref. ^44^). The samples were analyzed by flow cytometry on a FACSC alibur apparatus (FACS Calibur, BD, USA). Cells untreated were used as controls. Quantitation of cell cycle distributions was performed using Multi-cycle Software (ModFit software). The percentage of cells in G1, S, and G2 phases was calculated, respectively.

### Measurements of caspase-3 activity

Activities of caspase-3 were analyzed using a detection kit (Beyotime, China). Briefly, 2 × 10^6^ A375 cells were collected in a 2 ml tube and digested using trypsin. Supernatants were discarded after the cells were centrifuged at 4 °C (2000 × *g* for 5 min). The cells harvested were then washed once using PBS and lysed in 100 μl lysis buffer on ice for 20 min. The caspase-3 activities in the cell lysates were measured by mixing up the cell lysates with 40 μl of detection buffer and 10 μl of Ac-DEVD-pNA (2 mM), respectively, and incubating the mixtures at 37 °C for 120 min. The absorbance at 405 nm was monitored using a microplate Reader.

### Statistical analysis

All data are presented as means ± SEM in this work. Statistical significance was determined by using the one-way ANOVA for multiple comparisons, and the Student’s *t* test of the SPSS22.0 software for comparing the mean differences between every two groups. The data were considered to be significant when *P* < 0.05. All experiments were performed in triplicate.
